# Relationship Between Perceived Psychological Empowerment, Clinical Leadership, and Quality of Work Life Among Chinese Nurses: A Correlational Study

**DOI:** 10.1155/jonm/3643678

**Published:** 2025-04-14

**Authors:** Xiyan Gong, Cuicui Zhang, Yingyi Deng, Ying Zhong, Dongxia Liao, Qinying Jiang, Yuqin Chen, Changju Liao

**Affiliations:** ^1^Department of Nursing, Zigong First People's Hospital, Zigong, Sichuan, China; ^2^College of Nursing, North Sichuan Medical College, Nanchong, Sichuan, China

**Keywords:** clinical leadership, nurse, psychological empowerment, quality of work-life, structural equation model

## Abstract

**Aim:** To investigate the current state of work-related quality of life among Chinese nurses and to explore the mediating effect of clinical leadership between psychological empowerment and quality of work life.

**Background:** The quality of work-life significantly affects nursing team stability and the provision of high-quality care. Psychological empowerment and clinical leadership are considered influential factors in nurses' work-life quality. However, there is a lack of large-scale studies investigating the relationships between these variables.

**Methods:** This nationwide cross-sectional study utilized a multistage stratified proportional sampling approach to select 2633 registered nurses with more than one year of work experience from 17 tertiary general hospitals in China. The data were analyzed using descriptive analysis, Pearson correlation analysis, and structural equation modeling.

**Results:** The quality of work-life score for nurses was 3.38 ± 0.67. Positive correlations were observed between clinical leadership and quality of work life (*r* = 0.470, *p* < 0.01). Psychological empowerment was also positively associated with quality of work life (*r* = 0.570, *p* < 0.01). The structural equation model revealed that psychological empowerment had a positive direct effect on the quality of work life (*β* = 0.587, *p* < 0.001), with clinical leadership playing an intermediary role, accounting for 8.42% of the total effect.

**Conclusion:** Nurses' psychological empowerment was positively associated with the quality of work life and was partially mediated by clinical leadership. These results suggest that psychological empowerment and clinical leadership are key facilitators improving nurses' work-life quality.

**Implications for Nursing Management:** Hospital managers should further raise awareness of empowerment among nurse managers, implement scientifically sound empowerment and clinical leadership programs for nurses, enhance nurses' clinical leadership skills, and promote the quality of nurses' work-life.

## 1. Introduction

Nurses are a significant part of the healthcare system and are essential for protecting the health of patients, achieving universal health coverage, and facilitating the delivery of high-quality services [1]. With the rapid economic and social development, the evolution of the disease spectrum, and the acceleration of population aging, the shortage of nursing human resources, and high staff turnover rates have become universal reality problem in global public health [2, 3]. The World Health Organization reported that the global demand for nurses is expected to exceed 9 million by 2030 [4]. However, the current global nursing shortage is estimated to exceed 4.6 million [5]. This shortage of nurses not only affects the overall quality of healthcare services but also increases patient mortality, infections, and medication errors [6]. Additionally, it greatly increases the workload and pressure on nurses and can even affect their mental health. The data show that the nurse density in China is 20–29 per 10,000 population, which is significantly below the world average of 40–49 nurses per 10,000 population [4]. This indicates that Chinese clinical nurses are facing an overwhelming workload, which may jeopardize their quality of work life (QWL) [7]. The decline in nurses' QWL not only increases their job burnout [8] and willingness to leave [9] but also reduces their work motivation [10], ultimately affecting the quality of nursing care and patients' safety [6]. Additionally, it can damage the image and attractiveness of the nursing profession, making the nursing shortage even worse [11]. Therefore, how to improve the QWL and promote the development of effective nursing teams should become an important area of concern for nursing managers both domestically and internationally.

## 2. Background

QWL refers to the degree of personal needs of registered nurses being met while achieving organizational goals [12]. Previous studies have shown that nurses' QWL needs to be improved [10, 13, 14]. In a study conducted by Khajehnasiri et al. in Germany, the QWL of nurses was low, and only 5.7% of nurses had a good QWL [15]. In Turkey, the QWL among nurses is at a medium level, and factors such as long working hours and a high workload significantly affect the level [16]. In Ghana, nurses face resource scarcity and poor working conditions, leading to low levels of QWL for most nurses, with 50% expressing a desire to leave [9]. Similar phenomena have been observed in China. Many studies have shown that the QWL for Chinese nurses is currently at a low or moderate level [17–19]. China, as the world's most populous country, has only 2.9 nurses per 1000 population [4]. Many studies have shown that work-related stress is prominent among Chinese nurses [20]. As many as 42.8%–69.4% of Chinese nurses have expressed a willingness to leave their jobs [20, 21], and 74.8% of Chinese nurses report experiencing work-related stress [22]. With China's aging population and the opening of the three-child policy, the need for access to healthcare has increased. The working environment for Chinese nurses has become even more unsatisfactory, with hospital nurses being forced to work long hours, including more frequent night shifts, to cope with the high demand for patient care. The higher incidence of doctor–patient conflict and workplace violence has also adversely affected the QWL for Chinese nurses [23]. However, current research on QWL among Chinese nurses has focused on emergency department, intensive care unit (ICU), and oncology nurses, often with small sample sizes and a focus on analyzing the current status and influencing factors, with limited exploration of the mechanisms influencing nurses' QWL.

Psychological empowerment refers to the psychological experience nurses have when they feel authorized in their work [24]. As an intrinsic and continuous motivational factor, psychological empowerment helps nurses develop positive attitudes and behaviors [25, 26]. The influence of psychological empowerment on nurses can be observed in two main aspects. Firstly, as a protective factor, high levels of psychological empowerment enable nurses to experience a sense of psychological fulfillment, igniting intrinsic motivation, and enhancing work engagement [27], thereby increasing job satisfaction and improving the QWL [28]. Secondly, as an organizational support factor, psychological empowerment can motivate nurses to engage in clinical practice and decision-making by granting them greater decision-making authority and autonomy. While developing professional skills and decision-making abilities, nurses also enhance their clinical leadership [29]. Hence, psychological empowerment is not only a key factor in enhancing nurses' clinical leadership and job satisfaction but also a significant driving force for the sustainable development of the nursing profession.

Clinical leadership is the act of nurses providing guidance and support to patients and healthcare teams in their work practices [30]. As the focus of attention in the field of nursing management in recent years, nurses' clinical leadership plays an important role in ensuring patient safety, improving patient satisfaction, and promoting quality nursing care [31]. Previous studies have found that clinical leadership is an influential factor in nurses' QWL [32]. Better clinical leadership enhances nurses' professional competence, promotes their professional growth and development, and improves job satisfaction [10].

In summary, the factors that may influence nurses' QWL vary both domestically and internationally, but their suboptimal QWL is a common problem. Particularly in China, there is a serious shortage of nursing human resources [2]. It has also been found that nurses' QWL is related to a variety of individual, organizational, and family factors, such as nurses' age, marital status, educational background, individual health status, work treatment, and psychological elasticity [19]. Enhancing nurses' QWL is a multidimensional issue that requires comprehensive consideration of many factors. This includes not only providing a better work environment, scheduling work hours reasonably, upgrading salaries, and paying attention to nurses' physical and mental health but also attaching importance to nurses' individual growth and career development [33]. For example, a supportive team culture should be established. Authorizations should be conducted scientifically and reasonably, and nurses should be provided with necessary resources and training opportunities, so that they can enhance their sense of achievement at work and their personal capabilities [34]. These measures are crucial in enhancing nurses' QWL. So far, academic interest in nurses' QWL has been increasing. Perceived psychological empowerment [35] and clinical leadership [32] are both key factors in enhancing nurses' QWL. However, the mechanism of how psychological empowerment and clinical leadership specifically affect nurses' QWL is unclear.

The psychological empowerment theory suggests that when nurses feel that they have autonomy, influence, and competence at work, they are more likely to experience job satisfaction and an improved quality of life [25, 26]. It emphasizes the importance of stimulating employee potential and increasing job satisfaction in organizations. Psychological empowerment, as an intrinsic motivational mechanism, can help nurses enhance their positive feelings about their own work roles and the meaning of their work. This can improve their sense of professional identity and job satisfaction in the course of their dedicated work [36]. The conservation of resource theory (COR) argues that organizational support can improve employee satisfaction and well-being in the workplace. The theory emphasizes that employees with abundant resources tend to exhibit positive behaviors in order to maintain this favorable state, develop their skills, and prevent resource depletion. It is a classic theory that explains organizational behavior and employee resource acquisition in the past 30 years. The implications of COR for this research include two aspects. On the one hand, psychological empowerment, as a positive organizational support factor, can help nurses' access rich resources and promote their personal competence. On the other hand, when nurses perceive trust and support, they tend to be more motivated and creative, thereby promoting their ability for clinical leadership and job control and bolstering their sense of job satisfaction [37].

In conclusion, psychological empowerment from the organization and individual nurses' clinical leadership contribute significantly to nurses' QWL. However, to the best of our knowledge, there is no research on the relationship between nurses' clinical leadership, psychological empowerment, and QWL. Therefore, we empirically tested three hypotheses based on the psychological empowerment theory and COR:

• H1: The QWL is positively correlated with psychological empowerment.
• H2: The QWL is positively correlated with clinical leadership.
• H3: Clinical leadership partly mediates the relationship between psychological empowerment and QWL.

## 3. Methods

### 3.1. Study Design

A national multicenter cross-sectional survey methodology was used.

### 3.2. Participants and Setting

In this study, a multistage stratified proportional sampling method was used to select participants. Hospitals were sampled according to the distribution of tertiary hospitals in seven geographic divisions of China (East, South, North, Central, Southwest, Northwest, and Northeast). At the first stage, 17 hospitals were randomly selected from seven regions. The number of tertiary hospitals in each region was calculated based on data from the 2022 China Health Statistics Yearbook, ensuring that at least one hospital was sampled from each subregion, confirming a sampling ratio of 1/160. Thus, the hospitals sampled from each subregion include five in East China (773:160), two in South China (323:160), two in North China (358:160), two in Central China (357:160), three in Southwest China (479:160), one in Northwest China (218:160), and two in Northeast China (281:160). In the second stage, using the computerized random number method, five departments in each of the 17 hospitals were selected, for a total of 85 departments. Nurses who met the inclusion criteria in these 85 departments were used as the survey respondents. The inclusion criteria for this study were (1) being a Chinese registered nurse, (2) having at least 1 year of clinical nursing experience, and (3) providing informed consent and agreeing to voluntary participation in the study. The sample size for structural equation modeling (SEM) was calculated by the sample size calculator (https://wnarifin.github.io/ssc_web.html). We regard all variables as latent variables and calculate the degree of freedom to be 101. Utilizing a test level of *α* = 0.05, expected RMSEA = 0.04, and power (1 − *β*) = 80%, we considered 20% of invalid responses. Therefore, we need at least 318 participants. Since this study utilizes a web-based tool for data collection, we have appropriately increased the sample size to ensure the quality of the study. All nurses in the 85 departments who satisfied the inclusion criteria were surveyed.

### 3.3. Data Collection

Prior to the distribution of the questionnaire, 60 nurses were invited to pretest the questionnaire to evaluate the clarity and time required to finish it. Presurvey questionnaires were not included in the official survey results. During the formal investigation phase, data were collected via an online self-rating electronic questionnaire platform from February to April 2023. First, the nursing department of the surveyed hospital was contacted before the study began to inform them of the purpose and content of this study and to obtain their support and assistance. Nurses from the five randomly selected departments were invited by the director of the respective nursing department to participate in this survey, and the electronic questionnaire link or QR code was distributed to them through the WeChat platform. A uniform guideline was used to explain the purpose of this study, the method of completing the questionnaire, and any precautions. Respondents were informed that (1) participation in the study was voluntary and they could withdraw at any time during the study; (2) the questionnaire was completed anonymously; and (3) the results of the survey were used for scientific research only, so the information was kept strictly confidential. Subjects were asked to complete the questionnaire within 2 weeks, and each IP address could only be filled in the questionnaire once, and each question was set to require a mandatory answer. Of the 3500 questionnaires sent out, 867 were excluded as invalid (the questionnaires had an answer time of less than 240 s, or the answers given to all of the questions were the same), leaving 2633 valid questionnaires, with an effective return rate of 75.23%.

### 3.4. Measurement

#### 3.4.1. Demographics

The demographic characteristics studied included gender, age, marital status, educational level, position, working unit, years of working, work rank, night shift (per month), professional titles, regions, whether the respondent is a specialist nurse, whether the respondent is an only child, monthly income, and reasons for choosing nursing work.

#### 3.4.2. Psychological Empowerment

The Chinese version of the psychological empowerment scale (PES) was prepared by Li Chaoping [38]. The scale is widely used in the psychological field, including four dimensions measuring work significance, work efficiency, autonomy, and influence, with a total of 12 items. A five-level Likert scoring method is used to score 1–5 points from “*very strongly disagree*” to “*very strongly agree*,” with a total score of 12–60 points. A score of 1–2 points is considered a low level, 3–4 points is considered a medium level, and above four points is considered a high level. Cronbach's *α* coefficient is between 0.772 and 0.86 [38]. Cronbach's *α* coefficients of the four dimensions of work significance, work efficiency, autonomy, and work impact are 0.874, 0.729, 0.712, and 0.823, respectively.

#### 3.4.3. Clinical Leadership

The clinical leadership of nurses was measured by Patrick's clinical leadership scale (CLS) [30], which was developed in 2011 based on the transformational leadership theory and organizational empowerment theory. A five-point Likert scoring method is used to score 1–5 points, ranging from “*almost never*” to “*always*.” The higher the score, the higher the frequency of leadership behaviors listed in the scale, and the more effective the clinical leadership. The scale consists of five dimensions: challenging the status quo, sharing the vision, inspiring people, making people behave, and setting an example. Each dimension contains three items, totaling 15 items. This research utilizes the Chinese version of the CLS developed by Quan et al. [39]. The scale's Cronbach's *α* coefficient is 0.942, and each dimension's Cronbach's *α* coefficient ranges from 0.748 to 0.889. The Chinese version of the CLS has good reliability and validity and can be used as an effective tool to evaluate the leadership of clinical nurses in China [39].

#### 3.4.4. QWL

This research utilizes the Chinese version of the Work-Related Quality of Life Scale-2 (WRQoL-2) developed by Ya et al. [40], to measure the QWL of nurses. WRQOL-2 includes seven dimensions and 33 items, including work conditions, work pressure, work control, work evaluation, work–family balance, general well-being, and career satisfaction. A five-level Likert scoring system is adopted, with one point = “*very strongly disagree*,” two points = “*relatively disagree*,” three points = “*disagree*,” four points = “*agree*,” and five points = “*very strongly agree*.” The work stress dimension is scored in reverse, and the total score of the scale is the sum of factor scores divided by the number of factors [40]. The higher the score, the higher the work-related quality of life of nurses. The scale's Cronbach's *α* coefficient is 0.939, and each factor's Cronbach's *α* coefficient ranges from 0.652 to 0.859.

### 3.5. Ethical Considerations

Ethical approval was obtained from the Ethics Committee of Zigong First People's Hospital (Number: 78; Date: 7 December 2022). Participants submitting completed questionnaires were considered to have given informed consent. All data were collected anonymously, and respondents' confidentiality was protected.

### 3.6. Data Analyses

We used Excel and IBM SPSS 26.0 (IBM Corp., Armonk, NY, USA) for data entry and analysis. The QWL, psychological empowerment, and clinical leadership scores of nurses were tested for normality using quantile–quantile plots. All data were analyzed descriptively. The measurement data were described as means ± standard deviation, and the count data were described as frequencies and composition ratios (%). To explore the differences in QWL of nurses among different demographic groups, independent *t*-tests and analysis of variance were employed. Factors influencing nurses' work-life quality were verified using hierarchical multiple linear regression. SEM was used to analyze the factors that may be correlated with QWL. The calculations and structural model were executed using Mplus 8.3 to test the mediating role of clinical leadership in the relationship between psychological empowerment and QWL. *p* < 0.05 was regarded as significant in all two-tailed tests.

## 4. Results

### 4.1. Demographic Characteristics and Results of the Univariate Analysis

The 2633 nurses enrolled in this study had a mean age of 32.79 years (SD = 5.14), and 97.30% were female. Demographic characteristics and other participant information, as well as the result of *t*-test/ANOVA, are shown in Table 1. The results showed that there were significant differences in the scores on WRQoL-2 between age, years of working, department, technical title, position, employment status, number of night shifts, being only son or not, monthly salary, reasons for choosing nursing jobs, and regions (*p* < 0.05).

### 4.2. Correlation Analysis

As shown in Table 2, nurses' QWL was positively correlated with clinical leadership (*r* = 0.470, *p* < 0.01) and psychological empowerment (*r* = 0.570, *p* < 0.01). Nurses' clinical leadership was positively correlated with the level of psychological empowerment (*r* = 0.510, *p* < 0.01).

### 4.3. SEM for Factors Correlated With QWL

Mplus 8.3 was used to develop an SEM to investigate the mediating effect of clinical leadership on psychological empowerment and QWL. Using psychological empowerment as the independent variable, clinical leadership as the mediating variable, and work-related quality of life as the dependent variable, the parameters were estimated using the maximum likelihood method. The statistical measures included the chi-square test (*χ*^2^), comparative fit index (CFI), Tucker–Lewis index (TLI), root mean square error of approximation (RMSEA), and standardized root mean square residual (SRMR). As shown in Table 3, the confirmatory factor analysis results were *χ*^2^/*df* = 20.353 (*p* < 0.001), RMSEA = 0.076, 95% CI = 0.073– 0.079, TLI = 0.934, CFI = 0.945, and SRMR = 0.037. The larger dataset for *χ*^2^/*df* was due to the large sample size, while the other indicators almost met the requirements, and the model fit well.

In order to assess the significance of the mediatory effect, we employed the deviation correction Bootstrap method to calculate the confidence interval of the mediatory effect. We repeated the process and randomly selected 5000 samples from the original data. The results revealed that the path coefficient 95% CI was 0.016–0.092, and the interval did not include 0, indicating that clinical leadership acted as a mediator between psychological empowerment and QWL.

**Figure 1:**
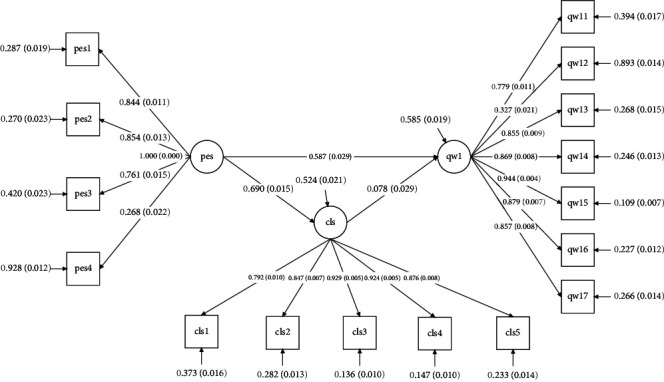
Model of the mediating role of CLS between PES and QWL. CLS, clinical leadership scale; PES, psychological empowerment scale; QWL, work-related quality of life scale-2.

The path coefficient diagram in Figure 1 and the results in Table 4 demonstrate that psychological empowerment has a direct and positive correlation with QWL (*β* = 0 0.587, *p* < 0.001), thereby confirming Hypothesis 1. Clinical leadership also has a direct and positive effect on QWL (*β* = 0.078, *p* < 0.001), thus confirming Hypothesis 2. Moreover, psychological empowerment has an indirect effect on QWL through clinical leadership (*β* = 0.054, *p* < 0 0.05), thereby confirming Hypothesis 3. The overall influence of psychological empowerment on QWL was 0.641 (*p* < 0.001), and the proportion of the mediating effect in the total was 0.054/(0.054 + 0.587) = 0.084.

## 5. Discussion

A national cross-sectional study based on SEM was conducted to explore the association between psychological empowerment, clinical leadership, and QWL among Chinese nurses in tertiary general hospitals. The results showed that the QWL of nurses was at a medium level. Age, years of work experience, number of night shifts per month, average monthly income, being an only child, reasons for selecting nursing work, regions, psychological empowerment, and clinical leadership were all found to be correlated with QWL. Clinical leadership partially mediated the relationship between psychological empowerment and QWL.

This study revealed that the QWL of nurses is at a moderate level, which is in line with previous research [32]. The work stress dimension had the lowest score (3.19 ± 0.76) among all dimensions and was below average. This could be attributed to the demanding diagnostic and treatment tasks carried out by tertiary hospitals, which have a large number of patients with relatively severe and complex conditions, as well as the difficulty of work and the high workload of clinical nurses [41]. Moreover, the majority of the surveyed nurses (84.81%) were aged between 20 and 39 years, the age group that is usually the primary workforce in clinical nursing positions. This age group often faces not only considerable work pressure but also the challenge of managing work and family responsibilities, such as raising children and caring for the elderly. Such work–family conflicts can increase the pressure on nurses and are correlated with poor QWL [42].

The results of the univariate analysis revealed that age, years of working, department, technical title, position, employment status, number of night shifts per month, average monthly income, being only son or not, the reason for choosing nursing work, and regions were important factors influencing nurses' QWL. It was observed that with the increase in age, working years, and titles, nurses' QWL was improved. This could be attributed to the fact that with the accumulation of life and work experience, their professional capabilities increased, allowing them to better adapt to the high-intensity working environment [43], thus leading to higher job satisfaction and improving their QWL. On the other hand, younger and less experienced nurses had lower working abilities and negative emotions, resulting in lower job satisfaction [44]. Additionally, it was found that the more night shifts nurses worked per month, the lower their QWL was. This could be due to the disruption of individual life routines and endocrine disorders caused by frequent night shifts [45], which often lead to sleep disorders and increased psychological stress and fatigue, thus reducing the QWL [46]. The QWL is also correlated with work treatment and employment methods. Research has revealed that the most important motivating factors for nurses to work are the basic needs such as a good salary and good work treatment [47]. Most of the tertiary general hospitals in China have implemented equal pay for equal work to promote workload and income equivalence. However, some disparities still exist. The phenomenon that the QWL of nurses hired is below that of staffed nurses still persists. The QWL for only children is higher than that of non-only children, which stands in contrast to the findings of Lina's study [48]. This discrepancy may be due to the fact that only children receive more family support, such as help with taking care of their own children and financial support, compared with non-only children, which helps to regulate the work-life conflict of nurses and reduces the general pressure of life. Among the reasons for choosing nursing, “personal aspirations” scored the highest, and “earning a living” scored the lowest. This may be due to the different professional identities between the two [27]. Those who chose nursing for “personal ideals” are more motivated to work, try to learn and master all of the skills that are needed for their work, and handle the difficulties of life and work with a more positive attitude. Conversely, when those who chose nursing to “make a living” feel that their reward does not match their effort, they are more likely to experience negative emotions and burnout, leading to a decrease in job satisfaction [49]. It was also found that the region may be correlated with nurses' QWL. The results showed that nurses' QWL is higher in the south and east regions and relatively lower in the northwest region. This may be related to the imbalance of economic development between different regions in China and the overall flow of nursing human resources to economically developed and resource-rich regions [50, 51]. This may lead to disparities in nurses' income levels, welfare benefits, and workloads between different regions, ultimately affecting their QWL.

The results of the Pearson correlation analysis indicated that psychological empowerment had a positive relationship with QWL (supporting H1). Boamah et al.'s study also confirmed the positive effect of psychological empowerment on nurses' QWL [52]. Psychological empowerment is an important influencing factor in nurses' QWL [53]. When nurses are given more decision-making power, they show higher job autonomy and self-efficacy and are better able to face difficulties with positive attitudes and behaviors [54]. At the same time, psychological empowerment can enhance nurses' perceptions of work meaning and work influence, and satisfy nurses' high-level needs, such as being respected, self-actualization, love, and belonging, which, in turn, enhance job satisfaction [53]. Additionally, effective clinical leadership is positively linked to QWL (supporting H3), as also suggested by Hong [32]. Good clinical leadership can reduce nurses' reliance on physicians [31], bolster their clinical decision-making ability and professional accomplishments [55], and create a better workplace atmosphere, thus increasing job contentment and QWL [56].

The SEM demonstrated that psychological empowerment has a direct correlation with nurses' QWL, and clinical leadership partially mediated the relationship between psychological empowerment and QWL (supporting H2). Nurses' clinical leadership had a direct and positive effect on patient safety, quality of care delivery, and nurses' personal job satisfaction [52]. Nurses with better clinical leadership were more inclined to adopt more positive and effective coping strategies, which, in return, had a positive effect on nurses' professional growth, career development, and personal well-being [32]. According to the psychological empowerment theory, only when managers create a supportive working environment for nurses and psychologically enhance nurses' experiences and perceptions of empowerment can empowerment be truly effective, stimulate nurses' intrinsic motivation, and enhance their personal competence and QWL [35, 57]. Appropriate psychological empowerment can give nurses more autonomy and self-confidence, access to more resources, information, and opportunities for learning and development [53], relieve their work stress [58], enable them to participate in their work and achieve their work goals in the most optimal way, improve their work efficiency and nursing service quality [54], as well as enhance their personal abilities and QWL. Psychological empowerment from managers can create a work environment conducive to learning and professional development for nurses and give them a platform to participate in decision-making and express themselves [35], which is conducive to the development of nurses' clinical leadership and enables them to achieve better professional development and more recognition, and their QWL will be enhanced accordingly [59].

In summary, psychological empowerment has a direct and positive effect on nurses' QWL and can also play an indirect role through clinical leadership. The psychological empowerment theory emphasizes the importance of providing organizational support to stimulate employee potential and increase job satisfaction [26]. When nurses feel empowered, they are more likely to improve their coping skills and ability to solve complex problems, which leads to increased engagement and satisfaction with their work, ultimately affecting the QWL.

## 6. Limitations

This study surveyed 17 hospitals in China to investigate the effects of psychological empowerment and clinical leadership on nurses' QWL. Despite the large sample size, there are some limitations to consider. For instance, the data were collected through self-report, which may introduce respondent bias. Additionally, the study only focused on nurses working in tertiary hospitals, which may limit the representativeness of the results. To increase the generalizability of the findings, future studies should also include nurses from primary and secondary hospitals. Moreover, this study only examined two factors that could be correlated with QWL, but there may be other factors that should also be taken into account. To gain a more comprehensive understanding of nurses' QWL, future studies should consider a wider range of factors.

## 7. Conclusions

This study used a multistage stratified proportional sample of 2633 nurses who completed an online questionnaire identifying demographic characteristics associated with nurses' QWL. In addition, this study explored the interrelationships and pathways of action between psychological empowerment, clinical leadership, and nurses' QWL. Psychological empowerment and clinical leadership were positively associated with nurses' QWL. Psychological empowerment was partially mediated by nurses' clinical leadership. These findings suggest that psychological empowerment and clinical leadership are essential factors in improving nurses' QWL. Hospital administrators and nursing leaders need to properly recognize the association between these variables in order to develop and enhance nurses' clinical leadership skills through proper empowerment and improve their QWL.

## 8. Implication for Nursing Management

Hospital managers should recognize the important role that QWL plays in maintaining the stability of the nursing team and promoting the quality of nursing safety and hospital development. In this study, we identified a number of demographic variables that may have a positive effect on nurses' QWL and that managers should consider when developing measures to enhance nurses' QWL. At the same time, this study identified the positive effects of psychological empowerment and leadership on nurses' QWL. Therefore, hospital administrators can develop interventions in both areas to improve nurses' QWL. On the one hand, they can learn from magnetic hospital management concepts, encourage clinical nurses to participate in decision-making, delegate scientific and reasonable authority to them, fully mobilize their work enthusiasm and autonomy, and promote the improvement of their QWL. On the other hand, a positive and supportive work environment can be created for nurses, while leadership training and career development opportunities can be provided to help them continuously improve their professional skills, effectively manage work-related stress, and ultimately enhance their QWL.

## Figures and Tables

**Table 1:** Univariate analysis of demographic information and quality of work life (*n* = 2633).

Item	*N* (%)	WRQoL-2 mean (SD)	*t*/*F*	*p* value
Gender			1.233	0.218
Male	71 (2.70)	3.48 (0.71)		
Female	2562 (97.30)	3.38 (0.67)		
Age (years)			4.628	0.003
20–29	960 (36.46)	3.33 (0.66)		
30–39	1273 (48.35)	3.40 (0.68)		
40–49	306 (11.62)	3.49 (0.65)		
≥ 50	94 (4.15)	3.40 (0.67)		
Marital status			2.057	0.128
Married	1883 (71.52)	3.40 (0.67)		
Singer	693 (26.32)	3.35 (0.68)		
Divorced or widowed	57 (2.16)	3.29 (0.54)		
Being only son or not			2.227	0.026
Yes	797 (30.27)	3.43 (0.69)		
No	1836 (69.73)	3.36 (0.66)		
Educational background			0.448	0.639
Junior college	644 (24.46)	3.39 (0.66)		
Undergraduate	1973 (74.93)	3.38 (0.67)		
Master' degree and above	16 (0.61)	3.52 (0.83)		
Years of working			2.704	0.029
1–5	802 (30.46)	3.34 (0.67)		
6–10	722 (27.42)	3.40 (0.67)		
11–20	811 (30.80)	3.38 (0.68)		
21–30	225 (8.55)	3.49 (0.64)		
≥ 31	73 (2.77)	3.46 (0.66)		
Department			6.218	< 0.001
Internal medicine	1062 (40.33)	3.41 (0.68)		
General surgery	773 (29.36)	3.36 (0.65)		
Obstetrics and gynecology	269 (10.22)	3.32 (0.66)		
Pediatrics	149 (5.66)	3.26 (0.69)		
Emergency	135 (5.13)	3.67 (0.69)		
ICU	157 (5.96)	3.30 (0.61)		
Operating room	88 (3.34)	3.41 (0.61)		
Technical title			4.201	0.015
Primary title	1623 (61.64)	3.36 (0.68)		
Middle title	841 (31.94)	3.40 (0.67)		
Advanced title	169 (6.42)	3.51 (0.63)		
Positon			3.703	0.011
None	2181 (82.83)	3.37 (0.68)		
Teaching nurse	128 (4.86)	3.39 (0.67)		
Quality control nurse	125 (4.75)	3.35 (0.73)		
Head nurse	199 (7.56)	3.53 (0.56)		
Employment status			−2.508	0.012
Contract	2077 (78.88)	3.37 (0.67)		
Permanent	556 (21.12)	3.45 (0.64)		
Night shifts (per month)			9.867	< 0.001
0	616 (23.39)	3.50 (0.70)		
1–5	749 (28.45)	3.40 (0.60)		
6–9	821 (31.18)	3.31 (0.67)		
≥ 10	447 (16.98)	3.33 (0.74)		
Monthly salary (RMB)			56.257	< 0.001
< 4000	641 (24.35)	3.21 (0.66)		
4000–5999	1097 (41.66)	3.35 (0.67)		
6000–7999	702 (26.66)	3.52 (0.64)		
≥ 8000	193 (7.33)	3.65 (0.62)		
Specialty nurses			−0.653	0.514
Yes	889 (33.76)	3.37 (0.67)		
No	1744 (66.24)	3.39 (0.67)		
Reasons for choosing nursing jobs			59.78	< 0.001
Personal ideal	491 (18.65)	3.68 (0.69)		
Making a living	1183 (44.93)	3.23 (0.63)		
Recommended by others	280 (10.63)	3.51 (0.64)		
Other	679 (25.79)	3.38 (0.64)		
Regions			4.618	0.032
East	659 (25.03)	3.54 (0.55)		
South	297 (11.28)	3.62 (0.56)		
North	294 (11.17)	3.35 (0.66)		
Central	319 (12.11)	3.37 (0.64)		
Southwest	531 (20.17)	3.50 (0.69)		
Northwest	206 (7.82)	3.26 (0.66)		
Northeast	327 (12.42)	3.33 (0.66)		

*Note:* WRQoL-2, work-related quality of life scale-2.

**Table 2:** Means, SDs, and correlations of all variables.

Scales and dimensions	Mean (SD)	1	2	3	4	5	6	7	8	9
1. WRQoL-2	3.38 (0.67)									
2. WCS	3.59 (0.78)	0.844^∗∗^								
3. SAW	3.19 (0.76)	0.433^∗∗^	0.246^∗∗^							
4. CAW	3.36 (0.75)	0.832^∗∗^	0.68^∗∗^	0.150^∗∗^						
5. HWI	3.38 (0.78)	0.842^∗∗^	0.674^∗∗^	0.274^∗∗^	0.750^∗∗^					
6. EEN	3.41 (0.83)	0.902^∗∗^	0.719^∗∗^	0.247^∗∗^	0.789^∗∗^	0.816^∗∗^				
7. GWB	3.31 (0.83)	0.865^∗∗^	0.654^∗∗^	0.305^∗∗^	0.705^∗∗^	0.738^∗∗^	0.822^∗∗^			
8. JCS	3.42 (0.81)	0.844^∗∗^	0.657^∗∗^	0.230^∗∗^	0.767^∗∗^	0.732^∗∗^	0.785^∗∗^	0.748^∗∗^		
9. CLS	4.08 (0.68)	0.470^∗∗^	0.427^∗∗^	0.196^∗∗^	0.414^∗∗^	0.367^∗∗^	0.450^∗∗^	0.384^∗∗^	0.417^∗∗^	
10. PES	3.78 (0.53)	0.570^∗∗^	0.476^∗∗^	0.237^∗∗^	0.491^∗∗^	0.468^∗∗^	0.538^∗∗^	0.526^∗∗^	0.492^∗∗^	0.510^∗∗^

*Note:* WRQoL-2, work-related quality of life scale-2; WCS, working conditions.

Abbreviations: CAW = control at work, CLS = clinical leadership scale, EEN = employment evaluation of nurse, GWB = general well-being, HWI = home–work interface, JCS = job and career satisfaction, PES = psychological empowerment scale, SAW = stress at work.

^∗∗^ *p* < 0.01 (two-tailed).

**Table 3:** Confirmatory factor analysis fits for the factors affecting quality of work-life.

Index	Fit index in the current study	Reference value
*χ* ^2^ (*df*), *p* value	2055.686 (101), *p* < 0.001	
Normed chi-square (*χ*^2^/*df*)	20.353	< 5
RMSEA (CI)	0.076 (0.073–0.079)	< 0.08
TLI	0.934	> 0.9
CFI	0.945	> 0.9
SRMR	0.037	< 0.08

*Note:* SRMR, standardized root mean square residual.

Abbreviations: CFI = comparative fit index, CI = confidence interval, RMSEA = root mean square error of approximation, TLI = Tucker–Lewis index.

**Table 4:** Direct, indirect, and total effects of the variables in the final model.

	Point estimate	Product of coefficients		Bootstrap				*p* value
				Percentile 95% CI		BC 95% CI		
		S.E.	*Z*	Lower	Upper	Lower	Upper	
Total								
PES ⟶ QWL	0.641	0.015	42.311	0.611	0.670	0.683	0778	< 0.001
Direct effects								
PES ⟶ QWL	0.587	0.029	20.459	0.530	0.643	0.599	0.739	< 0.001
Indirect effects								
PES ⟶ CLS ⟶ QWL	0.054	0.019	2.774	0.016	0.092	0.018	0.105	< 0.05

## Data Availability

The data used to support the findings of this study are available from the corresponding author upon request.
